# Human and Human-Interfaced AI Interactions: Modulation of Human Male Autonomic Nervous System via Pupil Mimicry

**DOI:** 10.3390/s21041028

**Published:** 2021-02-03

**Authors:** Catherine Spicer, Prashanna Khwaounjoo, Yusuf Ozgur Cakmak

**Affiliations:** 1 Department of Anatomy, School of Biomedical Sciences, University of Otago, P.O. Box 56, Dunedin 9054, New Zealand; catherinespicer27@gmail.com (C.S.); prash.khwaounjoo@otago.ac.nz (P.K.); 2 MedTech Core, Auckland 1010, New Zealand; 3 Brain Health Research Centre, Dunedin 9054, New Zealand; 4 Centre for Health Systems and Technology, Dunedin 9054, New Zealand

**Keywords:** sympathetic, parasympathetic, autonomic, virtual interaction partner, AI, HRV, pupil constriction, pupil dilation, mimicry, blush, gender effects

## Abstract

Pupillary alterations in virtual humans induce neurophysiological responses within an observer. Technological advances have enabled rapid developments in artificial intelligence (AI), from verbal systems, to visual AI interfaces with the ability to express, and respond to emotional states of a user. Visual AI interfaces are able to change their physical parameters, such as pupil diameter. Pupillary changes can alter heart rate, however, effects on heart rate variability (HRV) are unknown. HRV, is an autonomic, non-conscious parameter which monitors sympathetic and parasympathetic nervous system (PNS) activity. N = 34 male participants aged between 19–33 were subjected to a number of conditions such as pupil dilation, constriction and blushing. The present research is the first to investigate the effects of virtual human interactions on human HRV. Outcomes of this study were obtained using eye tracking and HRV measurements. Pupil dilation relative to constriction presented in the female virtual partner induced a significant right pupillary diameter increase (*p* = 0.041) in human observers. Additionally, female virtual partner pupil constriction relative to dilation induced a significant increase in participants’ PNS HRV response (*p* = 0.036). These findings indicate the ability of a female virtual interaction partner to modulate parasympathetic autonomic functioning in young healthy male humans. This allows first insights into the effects of interacting with virtual AI interaction partners, on human autonomic functioning, and may aid development of future virtual humans, and their implementation into relevant clinical settings.

## 1. Introduction

Artificial intelligence (AI) describes a machine operating with human-like qualities [1]. AI systems are rapidly advancing, from verbal based systems, such as Apple’s Siri, and Amazon’s Alexa, to the development of visual interaction partners with profoundly adapted interactive abilities [1]. In 2018 a New-Zealand company ‘Soul Machines’ released a digital human called Nadia [1]. Virtual human avatar interfaces such as Nadia have the ability to alter their physical parameters, such as pupil size [1]. Pupil size and blush are salient visual expressions of sympathetic activity, and emotional arousal, controlled by the autonomic nervous system (ANS) [2,3]. These expressions aid human interactions by indicating how an interaction partner is feeling, therefore indicating how an observer should appropriately respond [2]. Human-computer interaction studies found observers to mimic pupillary changes in a virtual partner, this enhanced trust, and affected subsequent decisions made by human observers [4,5]. Blush and pupil dilation are mediated by the sympathetic nervous system (SNS), and when presented in a virtual interaction partner enhance trust, and attractiveness ratings by both men and women [6,7,8].

Literature has further found manipulations of pupil size in virtual partners to alter human heart rate in an observer [8]. A gap in the current literature is knowledge of how these manipulations affect heart rate variability. Pupil size, cheek colour, and heart rate variability (HRV) are mediated by the ANS, a non-conscious system regulated by parasympathetic (PNS) and SNS branches [9]. Heart rate variability indicates variations in cardiac contractions timing, to suggest which branch of the ANS is dominating at a given point in time [10]. Trust is promoted by salient visual cues, such as increase in pupil size, and cheek colour [6,7,8]. If such cues elicit changes in HRV, this enables virtual partners to non-consciously manipulate human physiological functioning. It is important to determine whether virtual interaction partners possess the ability to modulate human pupil size and HRV, which regulates human emotion [3,5]. If changes in pupil size and cheek colour presented in a virtual partner induce subsequent changes in human HRV, this indicates that virtual interaction partners have the ability to non-consciously modulate human autonomic functioning; and emotional regulatory of how calm, or excited an individual is. This pertains to their subsequent trust and decisions as a result.

The present study investigated the effects of manipulations in virtual partners on human observers autonomic functioning. This research is the first study to investigate communicative effects of virtual humans on autonomic functioning in humans through the incorporation of heart rate variability measures, additionally being the first to incorporate stimuli consisting of full faces in colour, and a blush effect. The aim of the present research was to determine the effects of pupil size, and cheek colour manipulations of a virtual partner, on human observer autonomic functioning, measurable in pupil diameter and heart rate variability stimuli responses. Our primary hypothesis proposed manipulations of pupil size and cheek colour in a virtual human interface to modulate pupil size and heart rate variability in an observer. With a secondary hypothesis predicting gender of the virtual partner to differentially affect the male participants’ response.

Overall this research aims to communicate the importance of understanding the physiological modulating abilities of virtual humans, during interactions. This research may also be applicable in clinical practice in inducing ANS changes to individuals with short term autonomic dysfunction, such as those who experience panic attacks, or to assist anxious patients in hospitals. 

Presented below are the methods and analysis utilized for this study, along with our novel findings and discussion. Further discussion into the applications/impacts of our study and future work are also provided. 

## 2. Materials and Methods

### 2.1. Study Population

Inclusion criteria for this research were healthy right-handed, male volunteers between the ages of 19–33 without a neurological or psychiatric history with normal-to-corrected vision. To mitigate confounds, exclusion criteria included any neurological disorder, regular medication, colour blindness and smokers.

The present study incorporated healthy young males with an age range of 19–33 years. This is to reflect inclusion in relevant literature [11] and control for differential autonomic nervous system activation inducing human heart rate variability fluctuations. Previous research found older individuals exhibit reductions in heart rate deceleration [12,13] and females exhibit HRV fluctuations dependent on their stage of the menstrual cycle [10]. Therefore, we restricted participant age range and sex to avoid associated age and hormonal confounds affecting the measured parameters [10]. 

Thirty-six participants who met the inclusion criteria were recruited from Dunedin, New Zealand. Two participants dropped out of the study following completion of the first session, consequently a total of 34 participants completed all four experimental sessions. Due to data loss throughout experimentation statistical analysis used results from 30, 29, and 33 participants. 

This research was approved by the University of Otago Human Ethics Committee H19/084 (Health) and experiments adhered to ethical guidelines and regulations. Participants were required to sign a consent form prior to experiments commencing and were informed of their freedom to withdraw at any stage. The study was a single-center study, this ensured a constant environment for participant experimental trials. 

### 2.2. Stimuli

**Figure 1:**
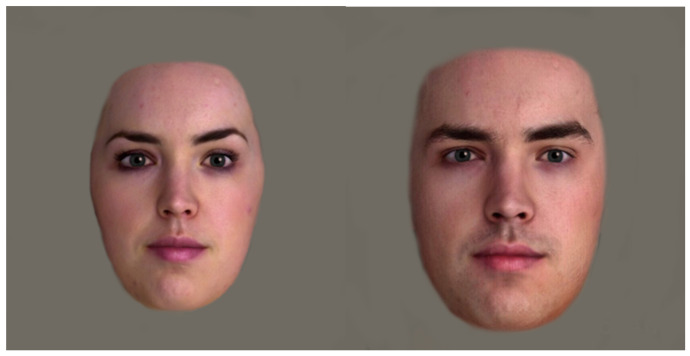
Female (**left**) and Male (**right**) Virtual Partner Stimuli—Full Face in Colour. Image stimuli was provided by Soul Machines.

Stimuli included videos of one male and one female virtual partner (VP), with neutral expressions, and light skin tone (Figure 1). Blush is less, or not distinguishable when presented in darker skin colourations [14]. As opposed to previous studies with stimuli derived from a facial expression set [5,8], ours were provided by Soul Machines, a New Zealand company which creates virtual humans. To produce the virtual human stimuli, Face App version 3.4.12 was used to overlay a male filter on the female face to create the male partner. This controlled for differentiations between the male and the female stimuli, ensuring that facial features were consistent. This method has not been used previously.

**Figure 2:**
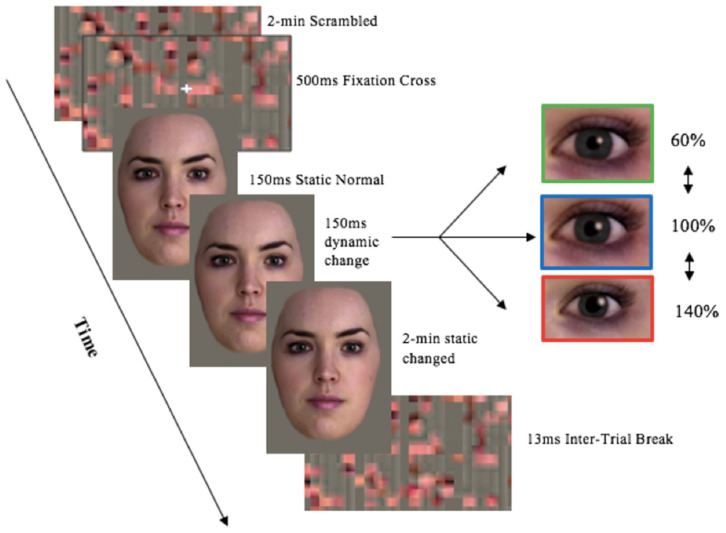
Experimental setup of Stream 1 stimuli presentation—Full Face in Colour pupil Manipulations trial proceeding. Image stimuli was provided by Soul Machines. Kret et al. [8] experimental set-up design was used as a proxy.

There were four unique experimental streams, with manipulations in; pupil size (Figure 2), cheek colour, pupils and cheeks, and grey eyes only. All presented virtual partners as a full face except the grey eyes only stream which was incorporated to reflect previous literature [11].

**Figure 3:**
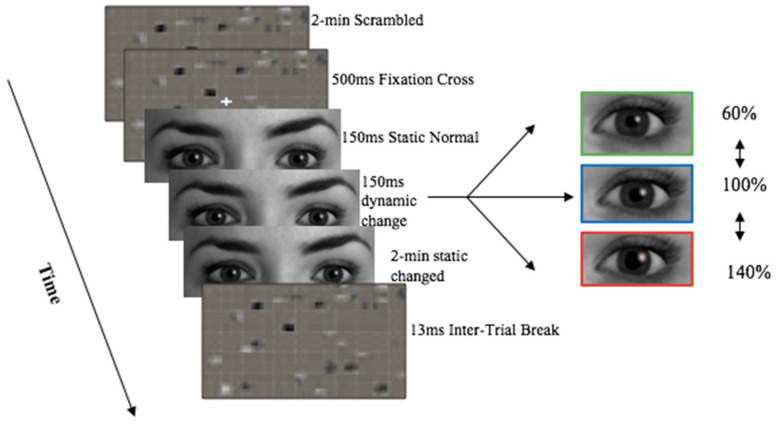
Experimental set up of stream 4 stimuli presentation—Grey eyes only pupil manipulations trial proceeding. Image stimuli was provided by Soul Machines. Kret et al. [8] experimental set-up design was used as a proxy.

**Stream 1:** Full face in colour with pupil manipulations: dilated, constricted, normal (Figure 2),
**Stream 2:** Full face in colour with cheek colour manipulations: red.
**Stream 3:** Full face in colour with manipulations of pupil size and cheek colour: pupils constricted-cheeks red, pupils dilated-cheeks red. The experimental control condition was the un-changed condition, where pupil size and cheek colour stays normal (100%).
**Stream 4:** Cropped eyes in grey scale pupil size manipulations: Normal, dilated, constricted (Figure 3).

Both the male and female virtual partner presented all 12 different stimuli conditions, equating 24 unique stimulus videos in total. Pupil manipulations included; pupil dilation (140% of the original size), constrict (60% of the original size), or remain static (100% normal). These manipulations reflect the physiological range of 3–7 mm [15], and reflects measures used in pupillary research [11]. Cheek colour manipulations included; cheek colour increase to pink (140% of original colour), red (180% of the original colour), or remain static (100% normal colouration/no blush). Blush manipulations reflected pupil manipulations due to no literature outlining the physiological range for changes in cheek colour. The male and female virtual humans were edited using GIMP 2.10, GNU image Manipulation Program (GIMP), The GIMP Development Team. (2019). GIMP. Retrieved from https://www.gimp.org. Iris colour was changed from brown to grey to control for luminance effects and ensure the pupil distinguishable from the iris. Pupil size was dilated 140% of original diameter, constricted 60%, or remained static. This produced a pupil size ranging from 3–7 mm which matches the physiological range in humans [5]. Cheek colour was also edited in. GIMP model-2.10 was used to create a red cheek condition. 

Scrambled images were created using the online image processing program G’MIC, model 2.7.1, manufactured by GREYC Lab (Caen Cedex, France, accessed 23 July 2019). Scrambled images were created for each of the four stimulus conditions. Scrambled images incorporated the same low level features stimuli, including luminance and contrast, thereby improving experimental control by being presented prior to stimuli to control for any difference in luminance, and avoid pupillary light reflex [2,5,11]. Using PowerPoint version 15.21.1, (Microsoft Office 2016); manufactured by Microsoft (Redmond, WA, USA). PowerPoint features including; effects, shrink/grow, and fade allowed for the pupillary changes and cheeks to occur dynamically (over time), as it would in a human interaction, as opposed to just having static images. Stimuli was presented in PowerPoint. The slides were timed to change to the appropriate stimuli presentation time.

### 2.3. Experimental Procedure

On the first visit, participants were provided the experimental proceedings and a consent form, participants also completed an online entry questionnaire, which included the Ishihara colour blind test, a test most commonly used, and regarded as the gold standard for rapid diagnosis of red-green colorblindness [16]. This ensured blush stimuli would be observable. Each experimental visit lasted 60 min, and consisted of four stimuli streams, each consisting of between 2 and 4 trials. 

At the start of each trial either a colored, or greyscale scrambled image made from a combination of stimuli faces, was presented for 2 min prior to stimuli to adhere to the minimum 1-min baseline recording required for heart rate variability [17]. A white fixation cross overlaid the scrambled image for 500 ms directing focus to the center of the screen where the virtual partner’s pupils would be displayed. Participants were instructed to look at the computer screen, and fixate on the eyes when presented. Static stimuli were presented for 1500 ms. Following this, manipulations to pupil size, cheek colour, or a combination of both cheek and eye manipulations dynamically over 1500 ms, or no manipulations at all (static) occurred. Pupil mimicry reaches its peak within 3000 ms [5], which was why 1500 ms of static face stimuli followed by 1500 ms change in stimuli was chosen. A final static presentation of the changed stimuli lasted 2 min to ensure an accurate recording time for heart rate variability [17]. The inter-trial break followed for 13 s, due to hemodynamic responses returning to baseline within 9300–12,300 ms [11]. A coloured scrambled image was presented during this time. There were a total of 12 trials per session.

### 2.4. Experimental Design

**Figure 4:**
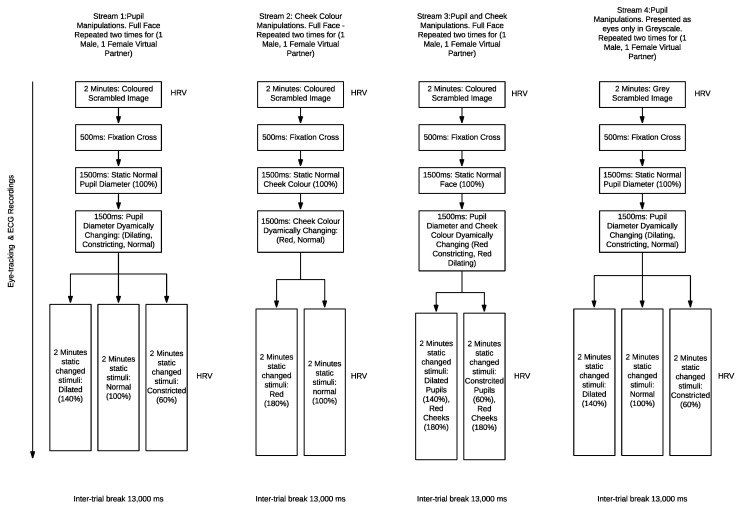
Flow chart of experimental design variables for the multiple streams, pupil alterations, cheek colour, pupil and cheek and greyscale cropped eyes.

The present research modulated pupils: dilated, constricted, normal; cheeks color: red, normal; virtual partner gender: male, female within a repeated measures within-participants design (Figure 4). All participants were exposed to all stimuli conditions in a randomized order which adheres accuracy due to participant being their own response comparison between stimuli types. This design was also applied in previous relevant studies [5]. Experimental stimuli presentation was randomised within, and between experimental streams 1–4 (Figure 4) to eliminate learning effects. Independent variables were virtual partner gender, cheek colour intensity, pupil size presented in a full coloured face, and pupil size in a cropped eye region in grey scale. The dependent variables included; participants’ pupil diameter and heart rate variability responses to stimuli. The control variables include the virtual partners with normal pupil size, and cheek colour. This was a single blind study, with participants blinded to the conditions. Windows of the experimental room were covered and blinds drawn to control for luminance effects. During the experiment the lights were switched off so the room was dark, and the only visible light was from the computer screen in which the stimuli were presented.

### 2.5. Physiological Measurement and Equipment

Pupil diameter, and heart rate variability were measured using an eye tracker; Tobii Pro Glasses 2 (Tobii Technology, Stockholm, Sweden) and Shimmer model 3 (Shimmer Sensing Ltd., Dublin, Ireland) electrocardiogram (Shimmer3 ECG), manufactured by Shimmer, and Realtime Technologies (Dublin, Ireland). Tobii glasses worn on the face, measured pupil diameter changes, and eye fixations. This enabled determination of stimuli effects on pupil size. The Tobii was calibrated to each participant by them fixating on a calibration dot, this ensured accuracy of eye tracking. The ECG, recorded through electrodes adhered to the participant’s chest in accordance with shimmer protocol. Participants sat 75 cm from the 27 inch Philips model: HW59220I Ultra HD Dell UltraSharp 24 computer screen, manufactured by Dell, Inc. (Round Rock, TX, USA). Participants faced the screen and were placed a certain distance away from it. This distance was applied in previous literature, and reflects informal communication between individuals, and ensures ecological validity of stimuli presented on the screen to be life size, and is suitable for eye tracking [5,11]. Recordings from each of the devices were collected in iMotions 8 programme. version 8, manufactured by iMotions, and Neuroelectrics (Copenhagen, Denmark). We then exported these recordings into Microsoft Excel and then imported onto Kubios HRV Premium Software (Ver. 3.3, www.kubios.com), manufactured by Kubios Oy (Kuopio, Finland). On the day of experiments, all data recorded digitally was transferred to the Principal Investigator’s computer with a password restricted access. Raw data in the monitoring devices were deleted.

### 2.6. Data Analysis

Pupil Mimicry, and eye tracking data was analysed by quantifying pupil diameter changes of the left and right eyes in response to 1.5 s of static normal stimuli, 1.5 s of dynamic stimuli presentation, and the whole two minutes of stimuli presentation. Heart rate variability data was analysed by comparing the response of the participant during the two minutes of normal static stimuli to the manipulated stimuli which dynamically changed. Shimmer 3 ECG enabled the collection of heart rate variability parameters. This was analysed using Kubios HRV analysis software, to produce outputs of the participants’ heart rate variability parameters; RMSSD, PNS, SNS, Stress index (SI). The PNS output was approximated, from an index (PNS index) that makes use of RMSSD, SD1 (short term heart rate variability), and mean RR. RMSSD is a time domain variable of heart rate variability, and describes the root mean square of the successive difference, therefore how much on heart beat varies in length from the next [18]. RMSSD is a common factor for PNS system activity. The SNS output was approximated from an index (SNS index) which makes use of mean HR, stress index, and SD2 (long term heart rate variability). An individuals’ SNS, and PNS indices are scaled based on the population, to provide a reliable measure of ANS activity [18]. The chosen HRV outputs were best suited to our experimental design in terms of feasible experiment duration, other frequency related HRV parameters require longer recording times in the order of hours and hence was not considered. In addition, the HRV parameters used above are some the most widely used [18,19,20,21,22] and Kubios ANS indexes and stress indices are being utilized to greater degree in studies [19,20,23,24,25,26,27,28] as good approximations of ANS activity. Overall these parameters enabled the inference of the effects of Male and Female Virtual interaction partner on the male participants autonomic functioning of the heart, and take into consideration the potential emotional contributions of human-virtual human interactions on HRV [23].

### 2.7. Statistical Analysis

**Figure 5:**
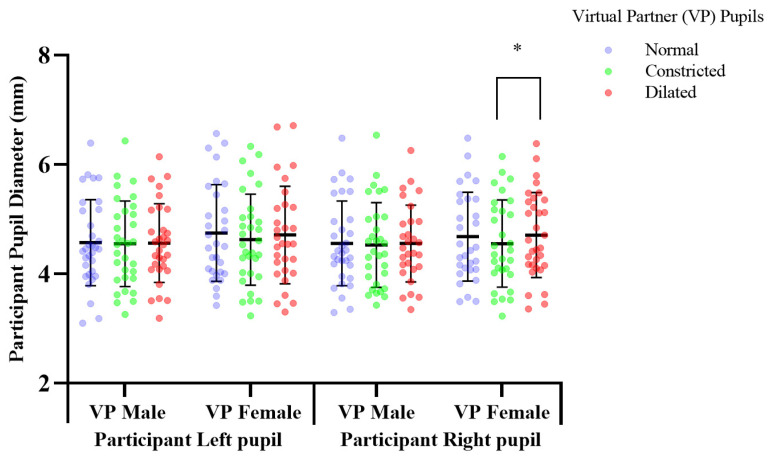
The effect of pupil manipulations of virtual partners (VP) presented as full face in colour on human observer pupil size. Participants’ (n = 30), right pupil diameter significantly increased * *p*, 0.041 over a two-minute duration, in response to observing a female virtual partner’s pupils dilating, compared to when the pupil diameter constricted.

**Figure 6:**
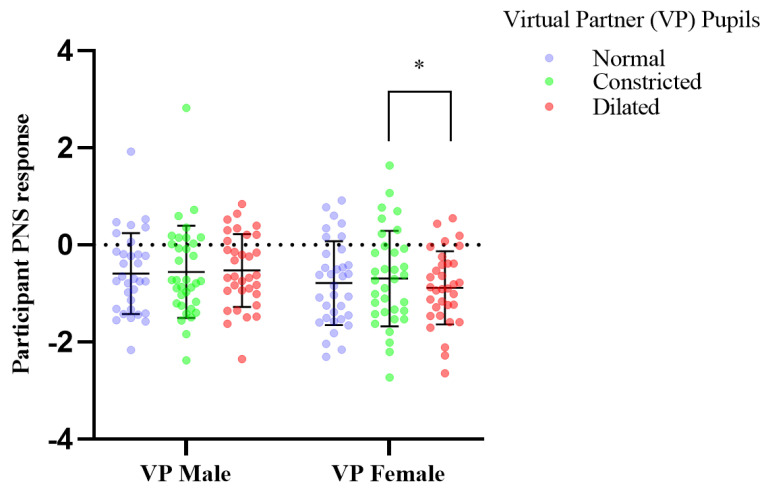
The effect of pupil manipulations of virtual partners (VP) presented as full face in colour on human observer heart rate variability PNS response. Participants (n = 33), mean parasympathetic (PNS) output of heart rate variability, recorded over two minutes showed significant increase in response to the female virtual partner’s pupils dynamically constricting; * *p*, 0.036; in comparison to the dilation of the female partner’s pupils.

**Figure 7:**
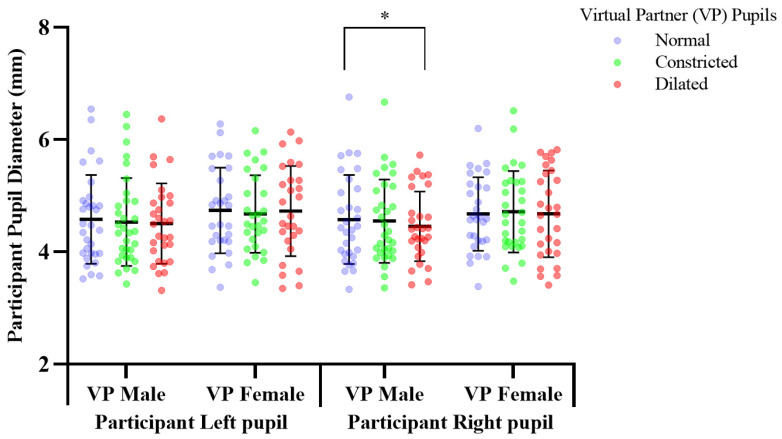
The effect of pupil manipulations of virtual partners (VP) presented as a cropped eye region in grey scale on human observer pupil size. Participants (n = 29), right pupil diameter significantly decreased; * *p*, 0.035 in response to observing a male virtual partner’s pupils dilating, compared to when the male virtual partner had a static normal pupil diameter.

All results presented were statistically analysed using Prism, model 8.2.1., by GraphPad Software Inc. (San Diego, CA, USA). The number of participants recruited was based on a power analysis as well as ensuring participant numbers were similar to that used in previous research with a similar experimental design which catered for a small effect size for pupil mimicry [5]. A repeated measures within—participants power calculation was performed which indicated a minimum of 28 participants were required to detect small effects at a power of 1 − β > 0.8, with a p-threshold of *p* = 0.05. We included an additional 6 participants to provide a power safety margin to ensure a power safety margin cover for non-responders and losses. Normality was confirmed with Shapiro-Wilks tests. and a two-way mixed model, multiple comparisons, ANOVA was performed. Tukey multiple comparisons test was used to determine significance with Bonferroni corrected *p*-values. Variables of mixed model analysis included; independent variables of virtual partners’ manipulations and participants’ dependant variables of HRV and pupillary response. The mixed model was performed due to some minimal lost data, as an alternative to a repeated measures ANOVA. These missing values are evident in Figure 5, Figure 6 and Figure 7 with participant numbers analyzed being n = 30, 33, 29, respectively. Data is presented in the form of mean ± standard deviation.

## 3. Results

A series of repeat experiments measured participants’ autonomic responses to manipulations of pupil sizes, cheek colors in a male, and female virtual partner. This enabled determination of modulating effects of virtual humans. The results presented here with will be separated into the multiple streams and stimuli specific groups.

### 3.1. Response to Pupil Manipulations of Virtual Partners Presented as a Full Face in Colour

Previous pupil mimicry research presented virtual humans, as cropped eye regions in greyscale [2,5,11]. We determined the pupillary response to virtual humans with a greater ecological validity by presenting them as a full face in colour. Mean change of participant pupil diameter in response to viewing virtual human pupils dilated vs. constricted; constricted mean, 4.554, SD, 0.797, dilated mean, 4.708, SD, 0.778. Participants’ right pupil diameter significantly increased by 0.153 mm (df = 29, f = 1.193, *p* = 0.041), over 2 min, in response to observing a female virtual partner’s pupils dilating from 5 mm–7 mm, compared to when the pupil diameter constricted; 5–3 mm (Figure 5). There was no significant difference in participants’ pupil diameter in response to constricting, or dilating of the male virtual partner’s pupils, compared to the normal pupils, which acted as a baseline comparison measure (Figure 5, Supplementary Table S1).

Mean participant PNS response to viewing virtual human pupils dilated vs. constricted changed from constricted mean −0.691, SD, 0.982, dilated mean, −0.882, SD, 0.753 Participants mean PNS output of heart rate variability, recorded over two minutes showed significant increase in activity in response to the female virtual partner’s pupils dynamically constricting (df = 32, f = 1.674, *p* = 0.036); compared to dynamic dilation of the female partner’s pupils, which induced a significant decrease in mean parasympathetic output of heart rate variability (Figure 6). Trends also show an increase in PNS activity of male participants in response to female virtual partner pupil constriction, comparative to normal, and a reduction in PNS activity in response to the female virtual partners’ pupil dilation when compared to when her pupils are unchanged (normal) (Figure 6, Table 1). In addition, no changes were observed in the other measured parameters of RMSSD, PNS, SNS, Stress index (Table 1).

### 3.2. Response to Pupil Manipulations of Virtual Partners Presented as a Cropped Eye Region in Grey Scale

Pupil manipulations of the virtual partners presented as eye regions in grey scale, elicited a significant response specific to the male virtual partner only (Figure 7, Supplementary Table S4). Mean change of participant pupil diameter in response to viewing virtual human pupils dilated vs. normal; dilated mean, 4.452, SD, 0.622, normal mean, 4.574, SD, 0.792.

Pupil dilation of the male virtual partner induced a significant decrease of 0.122 mm (df = 28, f = 3.293, *p* = 0.035), in participants’ right pupil diameter over 2 min, in response to observing the male virtual partner’s pupils dilating from 5–7 mm, compared to when the male virtual partner had a static normal pupil diameter of 5 mm, which acted as a baseline control measure (Figure 7). This showed male participants to not mimic the male partner, instead responding with an opposite autonomic response; pupil constriction (Figure 7, Supplementary Table S4). In contrast there was no significant effect (*p* > 0.05) on participants autonomic functioning (see Supplementary Table S8).

Participant response to the female virtual partner showed a trend during the 1.5–3 s in which her pupils were dynamically changing in size, with participants’ left and right pupil diameter showing mimicry. This trend in mimicry also occurred in response to the male virtual partner pupils dynamically changing (1.5–3 s), however was in participants left eye only (see Supplementary Table S4).

### 3.3. Response to Blush of Virtual Partners

Blush is a salient visual expression of sympathetic activity [2,3]. Previous literature found pupil mimicry of humans’ observing manipulations in a virtual partners’ pupils [4,5]. Therefore, we tested whether presentation of blush, would induce autonomic mimicry, measurable in the sympathetic response in both pupil size and heart rate variability. There was however no significant effect (*p* > 0.05) of the blush on participants autonomic functioning (see Supplementary Tables S2 and S6).

### 3.4. Response to Simultaneous Blush and Pupil Manipulations in Virtual Partners

Virtual human stimuli included a combination of cheek colour, and pupil manipulations. Blush and pupillary changes are both salient visual expressions of autonomic functioning [2,3]. Therefore, we wanted to determine the effect of the two autonomic expressions presented together, on the autonomic response in the observer. There was no significant effect (*p* > 0.05) of manipulations of both pupil size and cheek colour presented in a virtual partner on human observer autonomic functioning, in regards to observer pupil size, or heart rate variability (see Supplementary Tables S3 and S7 online).

## 4. Discussion

Evolving technology has resulted in realistic, and dynamic, virtual interaction partners, which possess the ability to manipulate their own salient visual communication features such as pupil diameter. Implementation, of such technology into areas such as banking [1], and potential implementations in clinical settings raises ethical concerns regarding their potential influence on human autonomic functioning. Previous research presented virtual human stimuli consisting of the eye region in grey scale derived from a human face bank. This holds little ecological validity regarding human interactions [2,5,11]. Such research found virtual partners modulate emotions, and trust in human observers [29]. An area which required further investigation were the effects of this technology on human HRV, which is the gold standard for measuring human autonomic functioning and has also been associated with stress and emotion [9].

The present study explored potential modulation of human autonomic function by dynamically manipulating salient visual features in virtual human partners; pupil size, and cheek colour. Measurements of these effects on participants’ autonomic pupil diameter and heart rate variability responses were achieved using eye tracking and electrocardiogram recording and analysis. This provided novel insights on the effects of pupil mimicry of virtual humans on HRV. Additionally, the incorporation of blush, and pupil manipulations in virtual human stimuli, created by ‘Soul Machines,’ a company which design virtual humans, enhanced the ecological validity of our experimental stimuli, comparative to previous research. Our investigations provide first evidence of female virtual partners’ ability to modulate mimicry in male observers’ and also is the first to observe a pupillary lateralization effect.

### 4.1. Right vs. Left Pupil Response

Previous literature analysed mimicry as a mean pupil diameter change of both eyes [5], reporting mimicry to occur in both [30]. The present study is the first to consider pupil mimicry using a distinct analysis specific to each eye. Pupil manipulations in virtual partners presented as an eye region in grey scale, and a full face in colour elicited a right dominant pupillary lateralization response, with pupil manipulations in both male and female virtual partners inducing a significant pupillary response in participants’ right eye only (Figure 5 and Figure 7, Supplementary Tables S1 and S4). This lateralization response may be attributed to the pupillary response axis; the Locus Coeruleus norepinephrine system which regulates differences in pupil diameter between both eyes [31], inducing lateralization in response to emotional images [32]. Differential activation of this system is a potential mechanism inducing pupillary emotional responses, and mimicry which stimulate the Locus Corelus to induce a lateralisation effect. Thereby causing differentiation in the diameter between left and right pupils. Our finding of lateralization indicates the right eye to adhere greater responsiveness to activation initiated by emotional stimuli. No previous pupil mimicry studies having reported lateralization in humans, or primates’. Future mimicry studies investigating this effect are needed for greater understanding.

### 4.2. Pupil Constriction vs. Dilation

To determine effects of virtual human pupil size on human autonomic functioning, virtual partners’ pupils were made to dynamically constrict, and dilate by 40% comparative to normal pupil diameter. Differences in pupil diameter between constriction and dilation conditions were 80%, adhering to the physiological range for pupillary changes [30]. Human observer pupillary and HRV responses, were measured. Previous mimicry studies reported a 40% difference between constriction and dilation of the virtual partners’ pupils sufficient to induce pupil mimicry [30], this conflicts with our findings of an 80% difference only to be sufficient. This may be attributable to previous studies analysing mimicry effects on participants as a mean of both pupils, rather than individually [30].

Virtual partners presented as a full face in color, induced a mimicry response to the female virtual partner only with a statistically significant difference in participants’ mean pupil size over two minutes in response to pupil dilation and constriction (Figure 5), (see Supplementary Table S1). This mimicry response was reflected in the participants PNS cardiac activity (Figure 6, Table 1). Pupil constriction of the female virtual partner induced a significant increase in the participants parasympathetic pupillary and heart rate variability PNS response, in comparison to dilation which induced a decrease (Figure 5 and Figure 6). Pupil constriction in the female virtual partner induced a reduction in participants’ right eye pupil diameter, indicating parasympathetic dominance, in combination with an increase in the PNS output of participants’ heart rate variability. This was expected for pupil constriction is parasympathetically controlled and communicates this activity to an observer [33]. Female Virtual partner pupil dilation induced a decrease in parasympathetic activity, which also was expected due to being sympathetically modulated [34].

Differences between dilated and constricted pupil conditions compared to the normal over two minutes was not statistically significant. However, there was a trend with pupil dilation of the female virtual partner inducing an increase in participants’ pupil diameter compared to normal, and constriction inducing a decrease compared to normal (Supplementary Table S1). This result is likely due to pupil dilation, induced by an increase in sympathetic activity and constriction caused by greater parasympathetic activity compared to the normal [33,34]. This trend in mimicry over two minutes was likely in response to the participants’ pupil dynamically changing over 1.5 s to mirror that of the virtual partner, as previous literature found pupil manipulations to affect participants trust, which was quantified by a decision they made subsequent to viewing dynamically changing pupils, indicating pupillary stimuli to induce effects which continue even after cessation of stimuli presentation [30].

Our findings show a female virtual partner, presented as a full face in colour, to modulate parasympathetic functioning of male humans, by dynamically manipulating pupil diameter. The autonomic response of participants’ pupils, and heart rate variability showed synchrony in their PNS response, indicating the manipulations in virtual interaction partners can modulate mimicry effects in humans (Table 1). There was no significant sympathetic (SNS), response in HRV of participants (Table 1). Heart rate variability enables insight into the contribution of parasympathetic and sympathetic activity relative to one another [30,35]. The sympathetic, and parasympathetic nervous system is part of one intricate system; the autonomic nervous system, which operates in a dynamic balance. Despite suppressing one another to maintain balance, these systems may act independently as well due to comprising distinct neural pathways [19]. This may explain this lack of sympathetic response. Therefore, when virtual partners’ pupils constricted, this induced an increase in participant PNS activity via mimicry, indicating the participant was in a more relaxed state due to the PNS, which is a resting system being dominant [35].

### 4.3. Grey Scale Cropped Region

Virtual human stimuli presented as a cropped eye region in grey scale was included, using our own unique stimuli to replicate methods used in previous research [2,5,11]. This was to confirm the reproducibility of prior research, and have a comparison model for responses to manipulations in a virtual partner when presented as a more ecologically valid model; full face in colour. Previous relevant literature reported pupil mimicry [2,5,11]. The present study found no significant mimicry effects (*p* > 0.05) when pupillary changes were presented in a virtual partner as a cropped eye region in greyscale on human observer heart rate variability and pupillary parameters (see Supplementary Table S8).

Our findings oppose the mimicry response reported in previous literature [2,5,11], where dilation of the virtual partner in fact reduced the pupil diameter (Figure 7). When presented as an eye region in greyscale the male virtual human induced a participant response which opposed mimicry, and the female virtual partner induced no significant participant response. When presented as a full face colour the male virtual partner induced no significant response, comparative to the female virtual partner which induced a mimicry response. This result may be attributable to the full face of the female being viewed as more pleasing, and trustworthy, when presented in colour inducing a significant mimicry response, as opposed to when the female was presented in greyscale which reduced ecological validity, likely reducing participant relatability to her and resulting in this presentation of the female partner being unable to induce a significant mimicry response [4].

Comparatively the lack of mimicry in response to the male virtual partner when presented in the full face in colour, and the induction of a significant response opposing mimicry when the male was presented as the cropped eye region in grey scale may have been due to the male being seen as competition or potentially threatening and this perception being enhanced when the male was presented as eyes only [5]. These ideas are further discussed under gender effects.

### 4.4. Blush

Blush is a sympathetically modulated emotional response; observation of an emotional response evokes such emotion in an observer [36]. Presentation of blush in a virtual partner, was hypothesised to modulate sympathetic activity in humans, inducing pupil dilation, and a decreased in heart rate variability. Furthermore, blush combined with dynamic pupil dilation was hypothesized to increase sympathetic modulatory responses comparable to that induced by blush alone due to displaying two visual stimuli, with sympathetic activity [33,37]. Blush presented in conjunction with pupil constriction enabled understanding of human responses to stimuli regulated by different branches of the autonomic nervous system with opposing functions. Pupil constriction is under parasympathetic mediation, blush; sympathetic. This additionally enabled insight of whether pupils or cheeks are more salient communications factors depending on participant’s response being parasympathetic, or sympathetically dominated.

Blush was presented in the virtual humans gradually over 1.5 s, as an 80% increase from the normal cheek colour and faded after 1–2 min to be consistent with dynamic pupillary changes presented. Blush induced no significant effects on participants autonomic functioning, as determined by pupil diameter, and heart rate variability responses (Supplementary Tables S2 and S6). This may be due to blush distracting from the pupils of the virtual partners, when presented with pupil manipulations, or may have made the virtual partner less relatable and therefore associated as an out group partner, which defines an individual that the participants’ associates as different from them self, humans do not mimic outgroup partners [4]. Alternatively, the lack of response to the blush may indicate blush to not be key a communicative cue. The timing of human blush presentation, in addition to the percentage of blush colouration has not been reported in literature, such research would benefit further understanding.

### 4.5. Male vs. Female/Gender Effects

A male and female virtual partner were incorporated to determine the effects of virtual partner gender on the quality of interactions with participants’, as quantified by participants’ autonomic responses as a secondary outcome we were investigating. The gender of the virtual partner elicited differential responses. Only the female virtual partner modulated a mimicry effect on male participants parasympathetic functioning, in both pupillary and HRV responses (Figure 5 and Figure 6), (see Table 1, Supplementary Table S1). Comparatively, the virtual male partner evoked an opposing significant autonomic response in the cropped grey scale images. These findings indicate the presence of a gender effect in response to the virtual partners. The greater mimicry response to the female virtual partner, may be attributable to male participants associating more with her [4]. Majority of participants verbalized preference for the female virtual interaction partner, describing her as less intimidating and easier to fixate on.

Pupil mimicry is absent in a competitive context, when competition is removed pupil mimicry is observed [5]. The absence of significant mimicry between the male participants, and male virtual partner in the present study may be due to such competition. These effects were likely in response to the gender of the virtual partner, opposed to virtual partner’s facial features, due to facial features being controlled for by presenting the same face for both male and female and overlaying it with a gender face filter using Faceapp (Face App version 3.4.12); manufactured by Wireless Lab, (Skolkovo, Russia). Previous literature presented cropped regions of male and female virtual partners face in grey scale, and found gender effects with a stronger mimicry response to pupil constriction in response to a partner of opposite sex, however, found no gender effects on pupil dilation mimicry [30].

Gender effects were not statistically significant; however, a trend was apparent with mimicry responses indicating male participant preference for female virtual interaction partners. Further research deducing reason for observed gender effects through inclusion of female, and homosexual participants, may further expand understanding of virtual human interactions and gender implications.

### 4.6. Limitations & Future Research

The present study included young healthy male participants to control for differential autonomic nervous system activation. Females and those of an older age demographic were excluded due to variations in HRV. HRV decreases with age, with older individuals exhibiting reductions in heart rate deceleration, attributed to a reduction in parasympathetic cardiac control [12,13]. Sex specific differences in females are due to their menstrual cycle which causes an increased HRV compared to males [10]. Research incorporating females, and an older age demographic would enable comparisons to responses measured in young male adults. Previous research has shown females to exhibit increased arousal responses to stimuli [32]. The effects of virtual partners on females would be of interest due to pupillary responses in humans affected by arousal [13]. Therefore, females may adhere larger responses to manipulations in virtual humans. Incorporation of an older age demographic would enable comparisons between generations, with individuals who grew up using technology and those who did not. This would determine whether these groups are differentially affected. Influences of an interaction partner depends on the susceptibility of the individual they are associating with [38]. Therefore, older generations who use technology less may be less susceptible to such effects.

Previous mimicry research included experimental trials seconds in length, those in the present study were 5 min, due to incorporating heart rate variability, which has not previously been incorporated in this field of research. Heart rate variability requires two minutes of baseline and two minutes of stimuli presentation to attain accurate RMSSD HRV measurements [17]. Stimuli presentation in the present study was randomized to control for fatigue affecting participants’ response. Participants varied in their ability to focus on the virtual partner, comparing autonomic responses of these participants would enable understanding of how attention to an interaction partner influences consequent responses.

Previous research presented virtual partners as a cropped eye region in grey scale. This made it implausible for the present research to present highly dynamic virtual partners. We presented stimuli as faces which dynamically changed, however, virtual humans presented in this context cannot completely replace face to face human interactions due to limitations in their comparable relatability. Replication of the present research in the context of virtual partners, who move and blink, or are presented in 3D would likely increase adherence to real life human interactions, enhancing ecological validity and potential relatability.

Overall our findings provide insights into the potential benefits, regarding both health, and communication. These findings are significant, as they can influence the development of future virtual humans to aid clinical applications. With further investigation, we envisage applications in clinical settings, which could potentially aid short, and long term autonomic nervous system health. Further investigations could lead to the development of short term applications which could induce an immediate calming effect, and long term changes to aid individuals affected with disorders in autonomic nervous system functioning. Additionally, these findings highlight the ongoing diversity in the development and application of virtual humans into everyday life making future human interactions with such virtual humans inevitable.

## 5. Conclusions

The present research communicates the importance of understanding the physiological modulative abilities of virtual humans, during interactions. This aids current literature by providing insight into the effects of such interactions on autonomic functioning, while incorporating relevant virtual human stimuli from the virtual human company; Soul Machines. This enhances ecological validity in comparison to stimuli applied in previous literature. Our principal conclusion was that female virtual interaction partners have the ability to modulate parasympathetic functioning in healthy male humans. We further concluded that blush presented in a virtual partner induced no modulatory effect on a human observer, however pupil diameter did, suggesting pupils to be a more dominant facial cue aiding communication responses. This study is novel, presenting the ability of virtual human interaction partners to modulate parasympathetic functioning in healthy male humans with female virtual partners having a greater influence in this effect. These findings lay the foundation for further investigations, including studying the longer term effects and applications.

## Figures and Tables

**Table 1:** Measured participant parameters of RMSSD, PNS, SNS, Stress index in response to pupil manipulations in male and female virtual partners presented as full face in colour (* *p* ≤ 0.05).

	Male						Female					
	Normal		Constricted		Dilated		Normal		Constricted		Dilated	
	Mean	SD	Mean	SD	Mean	SD	Mean	SD	Mean	SD	Mean	SD
RMSSD	35.037	12.278	34.379	14.237	37.366	14.039	35.101	16.917	37.230	19.852	37.992	24.870
SI	11.091	3.479	12.024	4.918	10.862	3.885	12.298	4.482	11.756	4.091	11.586	4.034
PNS	−0.589	0.835	−0.554	0.947	−0.525	0.751	−0.785	0.864	−0.691 *	0.982	−0.882 *	0.753
SNS	1.113	1.544	0.971	1.109	0.835	1.029	1.222	1.220	1.113	1.358	1.171	1.328

## Data Availability

The data presented in this study are available on reasonable request.

## Supplementary Materials

The following are available online at https://www.mdpi.com/1424-8220/21/4/1028/s1. Table S1: Human Autonomic Pupillary Response of Participants to Pupil Manipulations of Virtual Partners Presented as a Full Face in Colour. Table S2: Human Autonomic Pupillary Response to Blush in Virtual Partners. Table S3: Human Autonomic Pupillary Response to Blush and Pupil Manipulations in Virtual Partners. Table S4: Human Autonomic Pupillary Response to Pupil Manipulations of Virtual Partners Presented as an Eye Region in Grey-Scale. Table S5: Human Autonomic Heart Rate Variability Response to Pupil Manipulations of Virtual Partners Presented as a Full Face in Colour. Table S6: Human Autonomic Heart Rate Variability Response to Blush of Virtual Partners. Table S7: Human Autonomic Heart Rate Variability Response to Blush and Pupil Manipulations in Virtual Partners. Table S8: Human Autonomic Heart Rate Variability Response to Pupil Manipulations of Virtual Partners Presented as an Eye Region in Grey-Scale.
