# Supplementary material for: Human and Human-Interfaced AI Interactions: Modulation of Human Male Autonomic Nervous System via Pupil Mimicry
Source: Sensors (Basel). 2021 Feb 3;21(4):1028. doi: 10.3390/s21041028 (PMC7913357; doi:10.3390/s21041028)
Supplement: Supplementary file 1 [file sensors-21-01028-s001.pdf]

sensors

Article

Human and Human-Interfaced AI Interactions: Modulation of Human Male Autonomic Nerve System via Pupil Mimicry

Catherine Spicer [1], Prashanna Khwaounjoo [1,2] and Yusuf Ozgur Cakmak [1,2,3,4,*]

[1] Department of Anatomy, School of Biomedical Sciences; University of Otago; Po Box 56, Dunedin 9054, New Zealand; catherinespicer27@gmail.com (C.S.); prash.khwaounjoo@otago.ac.nz (P.K.)
[2] MedTech Core, Auckland 1010, New Zealand
[3] Brain Health Research Centre, Dunedin 9054, New Zealand
[4] Centre for Health Systems and Technology, Dunedin 9054, New Zealand
* Correspondence: yusuf.cakmak@otago.ac.nz; Tel.: +64-03-479-4030

Supplementary Tables S1-S4
Participant's pupillary response to stimuli conditions; (manipulations) of the virtual partner outcome variables pupil diameter over time.
L – denotes left pupil
R- denotes right pupil

Supplementary Tables S5-S8
Participants HRV response to; stimuli conditions (manipulations) of the virtual partner.
SI – Stress Index
SNS – Sympathetic Nervous system (mean heart rate stress index, and SD2)
SD2 = long-term HRV
SD1 = short-term HRV
PNS- Parasympathetic nervous system - calculated from RMSSD, SDI and mean R-R (length of heart beat)

Supplementary Table S1: Human Autonomic Pupillary Response of Participants to Pupil Manipulations of Virtual Partners Presented as a Full Face in Colour

	Male						Female					
	Normal		Constricted		Dilated		Normal		Constricted		Dilated	
	Mean	SD	Mean	SD	Mean	SD	Mean	SD	Mean	SD	Mean	SD
0 - 1.5 sec L	4.439	0.816	4.442	0.825	4.389	0.721	4.644	0.895	4.518	0.972	4.637	0.952
0 - 1.5 sec R	4.389	0.769	4.447	0.810	4.351	0.681	4.532	0.794	4.429	0.862	4.645	0.839
1.5 - 3 sec L	4.459	0.798	4.452	0.896	4.493	0.686	4.621	0.888	4.613	0.900	4.657	0.964
1.5 - 3 R sec	4.425	0.835	4.414	0.829	4.485	0.637	4.559	0.781	4.516	0.827	4.636	0.782
2 Min L	4.570	0.788	4.550	0.782	4.561	0.720	4.746	0.885	4.627	0.832	4.711	0.891
2 Min R	4.559	0.776	4.527	0.775	4.557	0.702	4.681	0.811	***4.554**	0.797	***4.708**	0.778

(* = P<0.05)

Supplementary Table S2: Human Autonomic Pupillary Response to Blush in Virtual Partners

	Male				Female			
	Normal		Red		Normal		Red	
	Mean	SD	Mean	SD	Mean	SD	Mean	SD
0 - 1.5 sec L	4.439	0.816	4.364	0.797	4.644	0.895	4.414	0.928
0 - 1.5 sec R	4.389	0.769	4.309	0.766	4.532	0.794	4.447	0.834
1.5 - 3 sec L	4.459	0.798	4.533	0.827	4.621	0.888	4.546	0.894
1.5 - 3 sec R	4.425	0.835	4.429	0.832	4.559	0.781	4.585	0.778
2 Min L	4.570	0.788	4.531	0.686	4.746	0.885	4.600	0.823
2 Min R	4.559	0.776	4.471	0.652	4.681	0.811	4.614	0.697

(* = P<0.05)

Supplementary Table S3: Human Autonomic Pupillary Response to Blush and Pupil Manipulations in Virtual Partners

	Male								Female							
	Normal		Red		Red Constricted		Red Dilated		Normal		Red		Red Constricted		Red Dilated	
	Mean	SD	Mean	SD	Mean	SD	Mean	SD	Mean	SD	Mean	SD	Mean	SD	Mean	SD
0 - 1.5 sec L	4.439	0.816	4.364	0.797	4.541	0.765	4.478	0.812	4.644	0.895	4.414	0.928	4.543	0.837	4.613	0.824
0 - 1.5 sec R	4.389	0.769	4.309	0.766	4.496	0.713	4.412	0.802	4.532	0.794	4.447	0.834	4.294	1.143	4.537	0.772
1.5 - 3 sec L	4.459	0.798	4.533	0.827	4.618	0.818	4.592	0.840	4.621	0.888	4.546	0.894	4.617	0.902	4.506	0.791
1.5 - 3 R sec	4.425	0.835	4.429	0.832	4.535	0.789	4.493	0.778	4.559	0.781	4.585	0.778	4.348	1.184	4.463	0.742
2 Min L	4.570	0.788	4.531	0.686	4.498	0.725	4.549	0.780	4.746	0.885	4.600	0.823	4.570	0.820	4.652	0.816
2 Min R	4.559	0.776	4.471	0.652	4.481	0.762	4.520	0.820	4.681	0.811	4.614	0.697	4.281	1.123	4.579	0.771

(* = P<0.05). Blush: Red; Pupil diameters: Constricted, Dilated; Normal: Pupils normal and no blush.

Supplementary Table S4: Human Autonomic Pupillary Response to Pupil Manipulations of Virtual Partners Presented as an Eye Region in Grey-Scale

	Male						Female					
	Normal		Constricted		Dilated		Normal		Constricted		Dilated	
	Mean	SD	Mean	SD	Mean	SD	Mean	SD	Mean	SD	Mean	SD
0 - 1.5 sec L	4.506	0.844	4.464	0.868	4.426	0.743	4.490	0.933	4.416	0.867	4.651	0.883
0 - 1.5 sec R	4.519	0.829	4.491	0.850	4.392	0.655	4.450	0.839	4.487	0.913	4.674	0.805
1.5 - 3 sec L	4.529	0.878	4.475	0.850	4.551	0.754	4.442	0.873	4.426	0.877	4.604	0.923
1.5 - 3 R sec	4.512	0.844	4.507	0.857	4.488	0.674	4.387	0.770	4.499	0.952	4.599	0.839
2 Min L	4.579	0.790	4.529	0.783	4.504	0.714	4.736	0.762	4.673	0.691	4.725	0.802
2 Min R	***4.574**	0.792	4.546	0.742	***4.452**	0.622	4.673	0.657	4.713	0.726	4.675	0.773

(* = P<0.05)

Supplementary Table S5: Human Autonomic Heart Rate Variability Response to Pupil Manipulations of Virtual Partners Presented as a Full Face in Colour

	Male						Female					
	Normal		Constricted		Dilated		Normal		Constricted		Dilated	
	Mean	SD	Mean	SD	Mean	SD	Mean	SD	Mean	SD	Mean	SD
RMSSD	35.037	12.278	34.379	14.237	37.366	14.039	35.101	16.917	37.230	19.852	37.992	24.870
SI	11.091	3.479	12.024	4.918	10.862	3.885	12.298	4.482	11.756	4.091	11.586	4.034
PNS	-0.589	0.835	-0.554	0.947	-0.525	0.751	-0.785	0.864	***-0.691**	0.982	***-0.882**	0.753
SNS	1.113	1.544	0.971	1.109	0.835	1.029	1.222	1.220	1.113	1.358	1.171	1.328

(*P = <0.05)

Supplementary Table S6: Human Autonomic Heart Rate Variability Response to Blush of Virtual Partners

	Male				Female			
	Normal		Red		Normal		Red	
	Mean	SD	Mean	SD	Mean	SD	Mean	SD
RMSSD	35.037	12.278	35.186	14.526	35.101	16.917	32.283	12.733
SI	11.091	3.479	12.041	3.992	12.298	4.482	11.857	3.399
PNS	-0.589	0.835	-0.596	0.786	-0.785	0.864	-0.879	0.761
SNS	1.113	1.544	0.907	0.982	1.222	1.220	1.171	1.201

(*P<0.05)

Supplementary Table S7: Human Autonomic Heart Rate Variability Response to Blush and Pupil Manipulations in Virtual Partners

	Male								Female							
	Normal		Red		Red Constricted		Red Dilated		Normal		Red		Red Constricted		Red Dilated	
	Mean	SD	Mean	SD	Mean	SD	Mean	SD	Mean	SD	Mean	SD	Mean	SD	Mean	SD
RMSSD	35.04	12.28	35.19	14.53	35.44	13.92	36.88	14.45	35.10	16.92	32.28	12.73	35.00	12.79	36.21	16.26
SI	11.09	3.48	12.04	3.99	10.78	3.25	11.51	4.49	12.30	4.48	11.86	3.40	11.34	3.51	11.96	4.29
PNS	-0.59	0.84	-0.60	0.79	-0.67	0.82	-0.64	0.78	-0.79	0.86	-0.88	0.76	-0.76	0.75	-0.76	0.79
SNS	1.11	1.54	0.91	0.98	0.80	0.95	0.93	0.93	1.22	1.22	1.17	1.20	1.05	1.08	1.20	1.24

(*P<0.05)

Supplementary Table S8: Human Autonomic Heart Rate Variability Response to Pupil Manipulations of Virtual Partners Presented as an Eye Region in Grey-Scale

	Male						Female					
	Normal		Constricted		Dilated		Normal		Constricted		Dilated	
	Mean	SD	Mean	SD	Mean	SD	Mean	SD	Mean	SD	Mean	SD
RMSSD	36.968	15.808	35.839	13.721	38.742	17.235	36.568	20.681	34.375	18.505	39.212	20.608
SI	11.103	3.651	11.390	3.724	10.673	3.810	11.773	4.446	12.104	4.321	11.053	4.182
PNS	-0.614	0.878	-0.656	0.751	-0.640	0.969	-0.607	1.149	-0.822	1.019	-0.602	0.975
SNS	0.943	1.024	0.888	0.920	0.872	1.064	1.129	1.270	1.247	1.341	1.016	1.319

(*P<0.05)
